# Comprehensive analysis of the expression profile and clinical implications of regulator of chromosome condensation 2 in pan-cancers

**DOI:** 10.18632/aging.204403

**Published:** 2022-11-27

**Authors:** Xuanxuan Li, Kuo Kang, Yuanhao Peng, Lin Shen, Liangfang Shen, Yangying Zhou

**Affiliations:** 1 Department of Oncology, Xiangya Hospital, Central South University, Changsha, Hunan 410008, China; 2 National Clinical Research Center for Geriatric Disorders, Xiangya Hospital, Central South University, Changsha, Hunan 410008, China; 3 Department of General Surgery, Xiangya Hospital Central South University, Changsha, Hunan 410008, China; 4 Hunan Key Laboratory of Precise Diagnosis and Treatment of Gastrointestinal Tumor, Xiangya Hospital Central South University, Changsha, Hunan 410008, China; 5 NHC Key Laboratory of Carcinogenesis, Cancer Research Institute and School of Basic Medicine, Central South University, Changsha, Hunan 410078, China

**Keywords:** RCC2, pan-cancer, prognosis, tumor microenvironment, immunotherapy

## Abstract

The Regulator of Chromosome Condensation 2 (RCC2) is an important gene that regulates mitosis and cytoplasmic division in the cell cycle. Although there have been reported in several individual tumors, an integrative analysis of *RCC2* and its clinical significance across diverse cancer types is poorly elucidated. In this study, we performed integrative bioinformatics analyses to profile the expression landscape and assess the prognostic value of *RCC2* in pan-cancers. Correlations between *RCC2* expression and tumor-infiltrating immune cells, tumor mutation burden (TMB), microsatellite instability (MSI), chemokine and their receptors were analyzed using TCGA, ESTIMATE algorithm, and TISIDB database. We also explored the potential molecular functions of *RCC2* through functional enrichment analysis and protein interaction networks. We discovered that *RCC2* was highly expressed in various tumor tissues and was closely associated with cancer prognosis. Different *RCC2*-associated immune infiltration patterns were exhibited in different tumor-infiltrating immune cells. In addition, the *RCC2* had a potential role in regulating the tumor immune microenvironment and the formation of cancer-associated fibroblasts (CAFs). Meanwhile, *RCC2* showed a significant correlation with TMB, MSI, chemokines and their receptors in different tumor types. The role of *RCC2* as a clinical therapeutic target was further revealed from the perspective of the immune microenvironment. In conclusion, *RCC2* is closely associated with tumorigenesis and cancer-immune infiltration, and could be a promising prognostic and therapeutic biomarker in diverse cancers.

## INTRODUCTION

Regulator of Chromosome Condensation 2 (RCC2, as known as TD60) is located at the 1p36 site and is a highly conserved member of the guanine nucleotide exchange factor (GEF) *RCC1* family [1]. The *RCC2* gene, as a component of the chromosomal passenger complex (CPC), plays a vital role in regulating mitotic and cytoplasmic divisions during the cell cycle [2]. It has also been shown that *RCC2* is a coordinating gene of the fibronectin-dependent adhesion signaling pathway and has a regulatory role in the maintenance of directional cell migration and cell spreading [3].

Recently, several studies have shown that the *RCC2* is participated in the pathophysiological processes of cancers in different ways. *RCC2* expression was shown to be elevated in breast cancer tissues and was related to an inferior prognosis in breast cancer patients, according to research by Chen et al. [4]. Elevated *RCC2* expression is closely linked to the advanced tumor metastasis and unfavorable prognosis in lung adenocarcinoma [5]. In hepatocellular carcinoma, increased expression of *RCC2* promoted HCC cell invasion and chemoresistance to cisplatin [6]. Furthermore, elevated *RCC2* was associated with an inferior prognosis in glioma patients and increased radioresistance in glioblastoma tumor cells through the activation of DNMT1 transcription [7]. In esophageal cancer, it has also been discovered that RCC2 accelerates cancer growth and invasion by boosting proliferation, transformation, and migration by inhibiting *SOX2* ubiquitination-mediated proteasomal degradation [8]. Although *RCC2* is currently considered as a potential biomarker in several malignancies, its clinical implications across different tumors remain unclear.

Despite the current proliferation of tumor-related treatment strategies available, there are still a large number of patients with advanced cancer who have limited options for treatment modalities. Immunotherapy is a treatment approach that overcomes the immune escape pathway by modulating the body’s immune system to activate the anti-tumor immune response. Currently, the combination of immunotherapy, surgery, chemotherapy, and radiotherapy is becoming the new treatment modality being explored in the immunotherapy era. The tumor microenvironment (TME) is one of the factors affecting the success of immunotherapy, so it is crucial to understand the targets related to tumor immunotherapy.

Currently, *RCC2* has only been reported in some specific tumors, and there is still no clear evidence of its regulatory role and molecular mechanism in pan-cancer. Meanwhile, poorly is understood the immunomodulatory function of *RCC2* in the immune microenvironment. In the current research, we comprehensively profiled the expression and function of the *RCC2* gene in pan-cancer through the public database, including TIMER, GEPIA2, UALCAN, cBioPortal, Kaplan-Meier plotter and TISIDB etc. We further evaluated the connection between *RCC2* gene expression and immune microenvironment across various tumor types. We found that *RCC2* was significantly overexpressed in the majority of tumors and may contribute to the development of cancers. Besides, the expression level of *RCC2* in pan-cancer was significantly associated with the cancer immune microenvironment. These findings highlight the significant role of *RCC2* in diverse cancers and provide an alternative therapeutic target for clinical utility.

## RESULTS

### *RCC2* expression levels across various cancer and normal tissues

**Figure 1:**
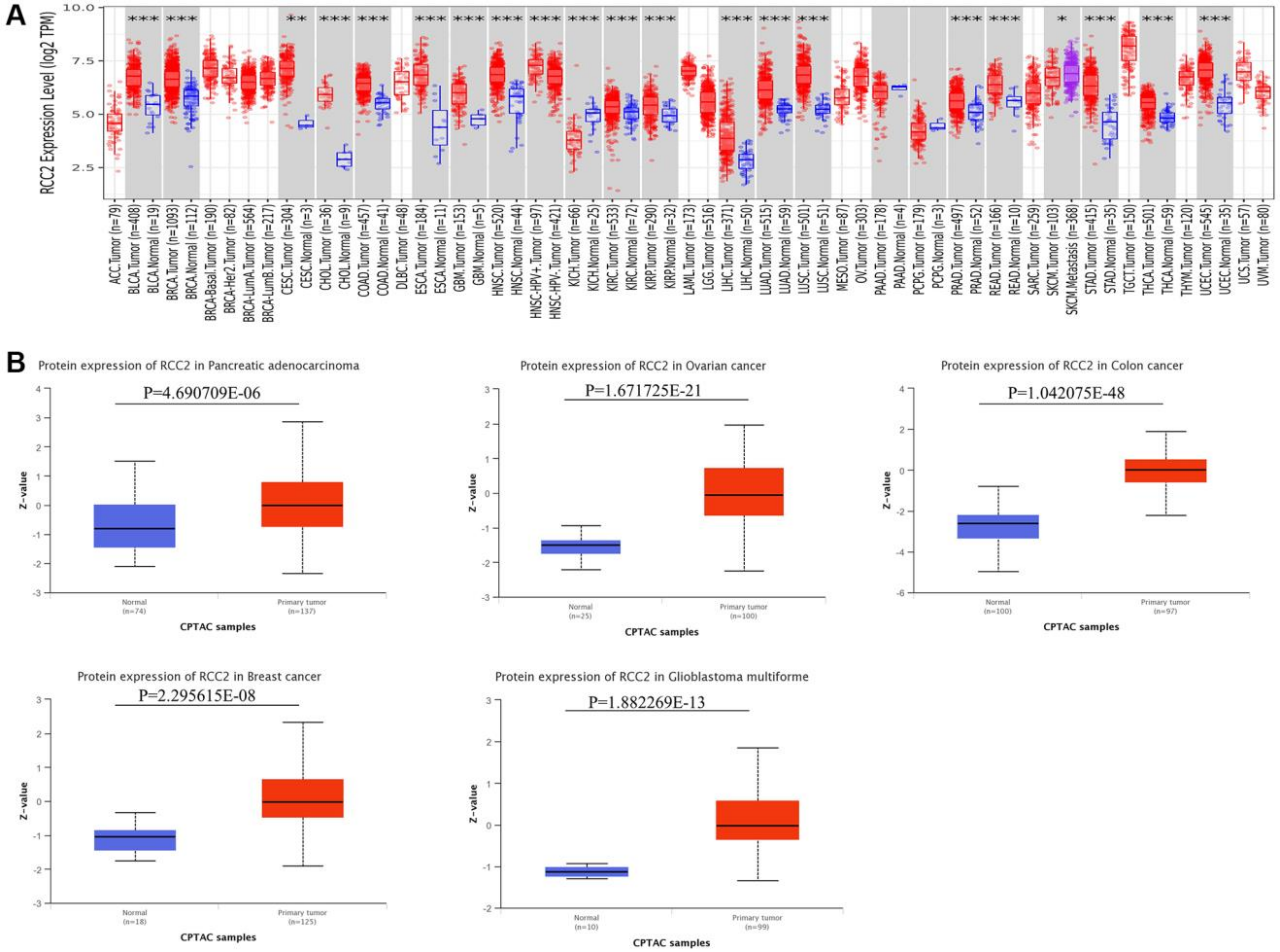
***RCC2* mRNA and protein expression in tumor and normal tissues.** (**A**) Expression levels of *RCC2* gene in tumor and normal tissues based on TIMER2.0 database. (**B**) The protein level of *RCC2* between normal and different tumor tissues. ^*^p < 0.05; ^**^p < 0.01; ^***^p < 0.001.

Firstly, we applied the TIMER2.0 website to evaluate the *RCC2* expression in pan-cancer. We revealed that *RCC2* was significantly overexpressed in the majority of tumor tissues, including breast invasive carcinoma (BRCA), cervical squamous cell carcinoma and endocervical adenocarcinoma (CESC), liver hepatocellular carcinoma (LIHC), esophageal carcinoma (ESCA), glioblastoma multiforme (GBM), NSC-HPV+, NSC-HPV−, bladder Urothelial Carcinoma (BLCA), head and neck squamous cell carcinoma (HNSC), kidney renal papillary cell carcinoma (KIRP), lung adenocarcinoma (LUAD), colon adenocarcinoma (COAD), kidney renal clear cell carcinoma (KIRC), prostate adenocarcinoma (PRAD), stomach adenocarcinoma (STAD), thyroid carcinoma (THCA), lung squamous cell carcinoma (LUSC), cholangiocarcinoma (CHOL), rectum adenocarcinoma (READ), uterine corpus endometrial carcinoma (UCEC). In contrast, only kidney chromophobe (KICH) demonstrated lower *RCC2* expression in tumor tissues (*p* < 0.05) (Figure 1A).

**Figure 2:**
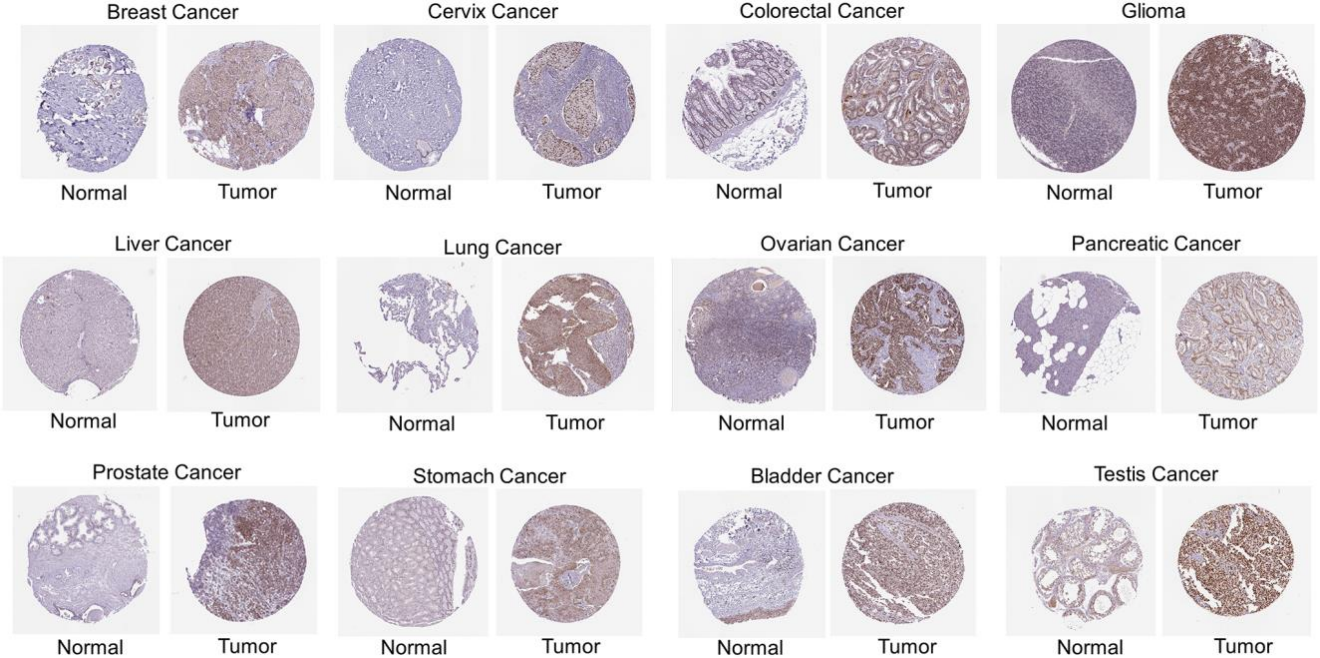
Immunohistochemical images of protein expression of RCC2 in various cancer types.

In addition to mRNA levels, we further analyzed *RCC2* at a protein level using the CPTAC dataset. The total protein expression of RCC2 was considerably elevated in pancreatic adenocarcinoma (PAAD), COAD, ovarian serous cystadenocarcinoma (OV), GBM and BRCA tumors compared to normal tissues (Figure 1B). We further validated the expression of RCC2 from proteomics data of 16 different cancer types [9]. We observed that RCC2 was overexpressed in BLCA, COAD, LUAD, LUSQ, LUSC, KIRC, PRAD, and STAD (Supplementary Figure 1). Besides, we also found that RCC2 protein was abnormally expressed in many cancer types from the HPA database. Figure 2 illustrated the immunohistochemistry findings regarding the expression level of RCC2 protein.

### *RCC2* expression with clinicopathological characteristics

**Figure 3:**
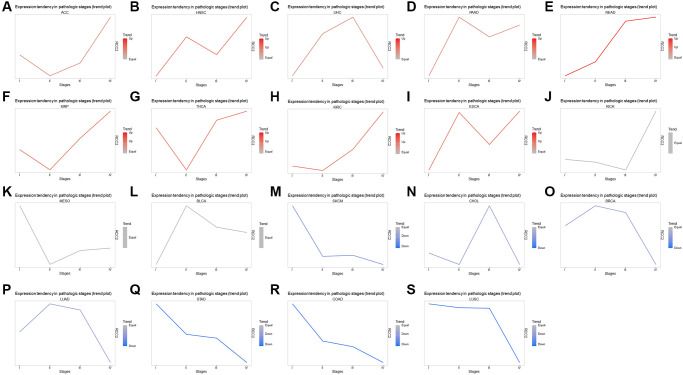
**Correlation with *RCC2* expression and clinicopathology.** (**A**–**S**) Trend plots summarize the *RCC2* mRNA expression trends from early to late stages in various tumor types.

To investigate the relationship between the mRNA expression of *RCC2* and clinicopathological features in pan-cancer. We utilized the GSCALite for the exploration of *RCC2* expression and the clinicopathological stage. The pathologic stage classified samples into Stage I to IV. The result revealed that *RCC2* expression has stage-specific expression changes in HNSC, KIRP, adrenocortical carcinoma (ACC), KIRC, and THCA (Supplementary Figure 2). The trend plot also demonstrated that the expression of *RCC2* in ESCA, KIRC, KIRP, HNSC, THCA, and READ gradually increased with increasing staging. However, *RCC2* mRNA expression in COAD, LUSC, STAD, and uveal melanoma (UVM) was negatively correlated with the stage (Figure 3).

**Figure 4:**
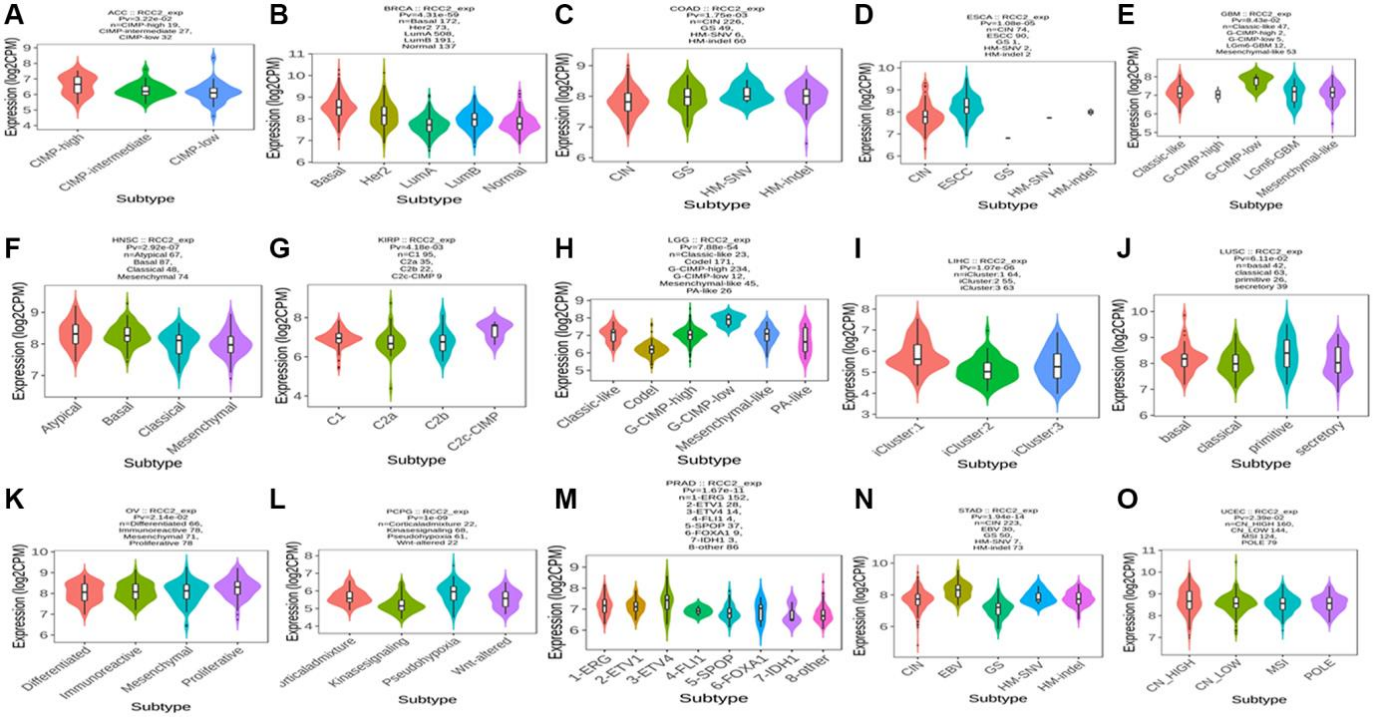
(**A**–**O**) The relationship between *RCC2* expression and various tumor molecular subtypes.

We then investigated the association between *RCC2* expression and molecular subtypes throughout pan-cancer using TISIDB’s “subtype” module. The findings presented that the molecular subtype of brain lower-grade glioma (LGG), GBM, ACC, BRCA, COAD, ESCA, KIRP, PCPG, PRAD, OV, HNSC, LUSC, STAD, LIHC, and UCEC were all strongly correlated with *RCC2* expression (Figure 4).

### Analysis of *RCC2* expression and survival in various cancer types

**Figure 5:**
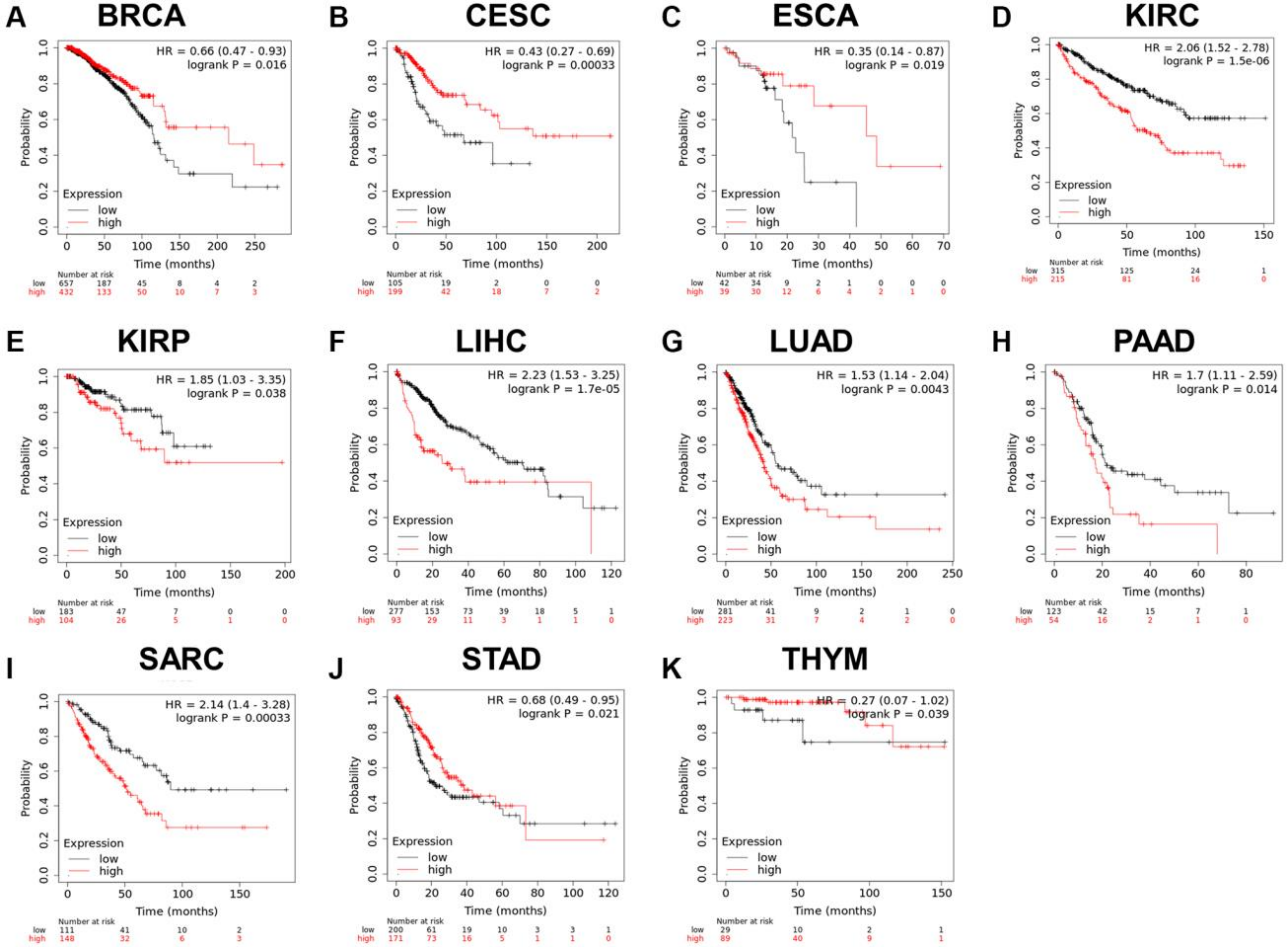
(**A**–**K**) Relationship between *RCC2* expression and overall survival analysis data in TCGA pan-cancer. Only tumor types with log-rank *p* < 0.05 were displayed.

Since the *RCC2* was overexpressed in numerous malignancies and was related to the tumor stage, we further explored its prognostic value in pan-cancers. The correlation between *RCC2* expression and survival endpoints such as OS and RFS was further analyzed using a KM plotter. We revealed that the elevated expression of *RCC2* was considerably related to an unfavorable prognosis in KIRC (HR:2.06, 95% CI:1.52–2.78, log-rank *p* = 1.5e-06), KIRP (HR:1.85, 95% CI:1.03–3.35, *p* = 0.038), LIHC (HR:2.23, 95% CI:1.53–3.25, *p* = 1.7e-05), LUAD (HR:1.53, 95% CI:1.14–2.04, *p* = 0.0043), PAAD (HR:1.7, 95% CI:1.11–2.59, *p* = 0.014), sarcoma (SARC) (HR:2.14, 95% CI:1.4–3.28, *p* = 0.00033); whereas, the high expression of *RCC2* was associated with a favorable prognosis in thymoma (THYM) (HR:0.27, 95% CI:0.07–1.02, *p* = 0.039), STAD (HR:0.68, 95% CI:0.49–0.95, *p* = 0.021), CESC (HR:0.43, 95% CI:0.27–0.69, *p* = 0.00033), ESCA (HR:0.35, 95% CI:0.14–0.87, *p* = 0.019), BRCA (HR:0.66, 95% CI:0.47–0.93, *p* = 0.016) (Figure 5). Furthermore, the high expression of *RCC2* was found to result in a shorter RFS in patients with KIRP, LIHC and PAAD (Supplementary Figure 3).

### Genetic alterations of *RCC2* in pan-cancers

**Figure 6:**
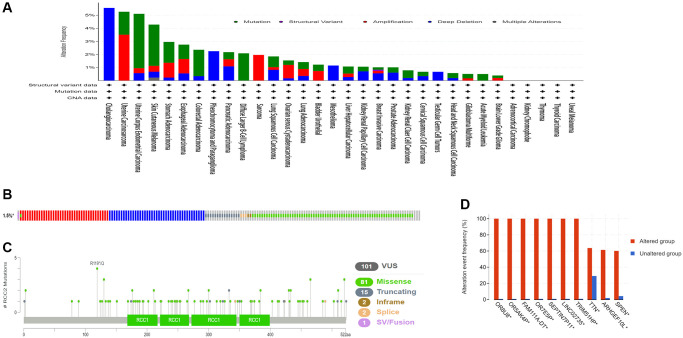
**The genetic alterations characteristic of *RCC2* in pan-cancers.** (**A**) Mutation type and frequency in all TCGA tumors. (**B**) Summary of *RCC2* structural variant, mutations, and copy-number alterations. (**C**)The site of different mutation types of *RCC2*. (**D**) The related genes alteration frequency in the *RCC2* altered group and unaltered group.

We then examined the genetic alterations of *RCC2* in pan-cancers by using the cBioPortal database. Our results demonstrated that the highest frequency type of *RCC2* alteration occurs in Cholangiocarcinoma, with “deep deletion” as the predominant type (>5%) (Figure 6A). Deep deletion was the only altered type in Cholangiocarcinoma (5.56%), Pheochromocytoma and Paraganglioma (2.25%), Mesothelioma (MESO) (1.15%), and Testicular Germ Cell Tumors (0.67%) (Figure 6B). Furthermore, we identified 101 mutation sites between amino acids 0 and 522, including 81 missense, 15 truncating, 2 in-frame, 2 splices, and 1 fusion mutation (Figure 6C). The genetic alterations in *OR8U8*, *OR5AK4P*, *FAM111A-DT*, *OR7E5P*, *SEPTIN7P11*, *LINC02735*, *TRIM51HP*, *TTN*, *ARHGEF10L*, and *SPEN* were more prevalent than in the altered group (Figure 6D).

### Relationship of *RCC2* expression with immune cell infiltration

**Figure 7:**
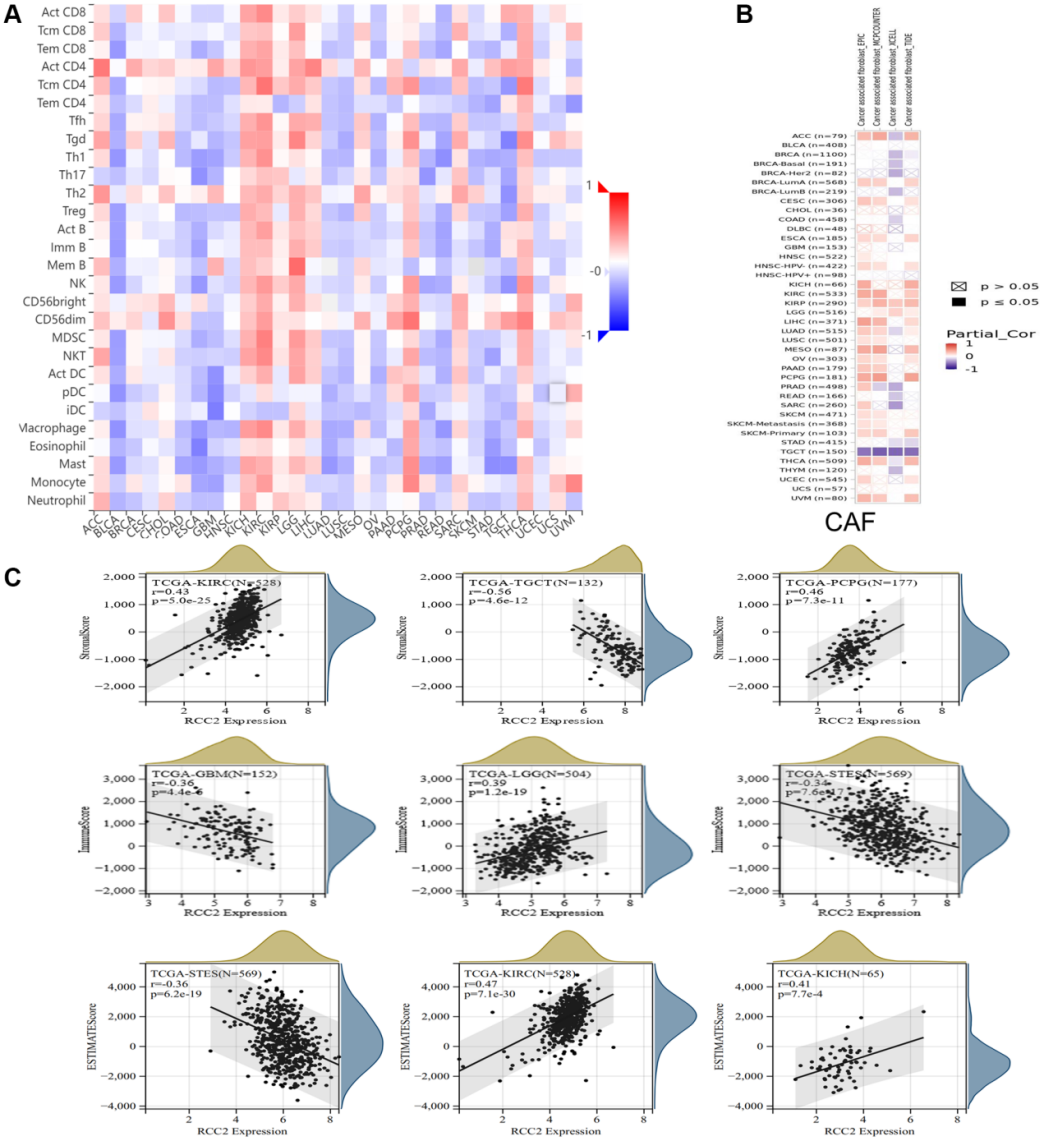
**Relationship between *RCC2* expression and immune microenvironment.** (**A**) The correlation heat map shows the relationship between *RCC2* expression and immune infiltrating cells. Red color represents positive correlation, and blue color represents negative correlation. (**B**) The relationship between *RCC2* expression and CAFs. (**C**) The top three cancer types with the most significant correlation between the degree of immune infiltration and *RCC2* expression were displayed.

Immunotherapy has emerged as a vital strategy for tumor treatment and has shown remarkable clinical effects. Therefore, we further investigated the immune microenvironment of tumors and the polymorphism of immune-related genes in immunotherapy. We investigated the association of immune cell infiltration and *RCC2* expression by using the TISIDB database. The clustering heatmap illustrated that *RCC2* expression was positively related to immune cells in KICH, KIRP, KIRC, LGG, thyroid carcinoma (THCA), and pheochromocytoma and paraganglioma (PCPG) tumors (Figure 7A). We further explored the “Gene” module of the TIMER2.0 database. The *RCC2* gene was positively related to cancer-associated fibroblasts (CAFs) in KIRP tumors while was inversely correlated to CAFs in testicular germ cell tumors (TGCT) tumors. (Figure 7B).

The immune and stromal scores of cancer tissues were calculated using the ESTIMATE method, and the three tumors with the most significant correlation between the scores were selected separately. We finally observed that *RCC2* expression was significantly associated with immune scores in 21 tumor types (Supplementary Figure 4) and was related to the stromal scores in 23 cancer types (Supplementary Figure 5). Among them, the most significant correlations were found between *RCC2* expression and immune scores in LGG, GBM, and Stomach and Esophageal carcinoma (STES). For stromal scores, there was a significant correlation between KIRC, TGCT, and PCPG. In KICH and KIRP, *RCC2* expression was positively correlated with ESTIMATE score, whereas it had negative correlation in STES (Figure 7C). We further analyzed the expression levels of *RCC2* among immune subtypes in pan-cancers and we found a significant correlation between *RCC2* and C1 (wound healing), C2 (IFN-gamma dominant), C3 (inflammatory), C4 (lymphocyte depleted), C5 (immunologically), and C6 (TGF-b dominant) in ACC, UCEC, TGCT, STAD, SARC, THCA, LGG, MESO, PAAD, LUSC, LUAD, KIRP, BLCA, ESCA, HNSC, KICH and KIRC (*p* < 0.05, Supplementary Figure 6). To further evaluated the efficacy of immunotherapy in patients with different expression levels of *RCC2*, we calculated the immunophenoscore (IPS) of patients with pan-cancers. We observed that *RCC2* expression was negatively associated with the IPS score among thirteen different cancers, while it was positively associated with the IPS score in OV (Supplementary Figure 7).

Additionally, utilizing the TISIDB database, the association between *RCC2* expression and immunotherapy were further analyzed, and statistically significant expression differences were only found between responders and non-responders in uroepithelial tumors (*p* = 0.00133, Supplementary Table 1). However, no statistically significant differences in mutations were seen between responders and non-responders.

### Correlations between *RCC2* expression and checkpoint gene markers

**Figure 8:**
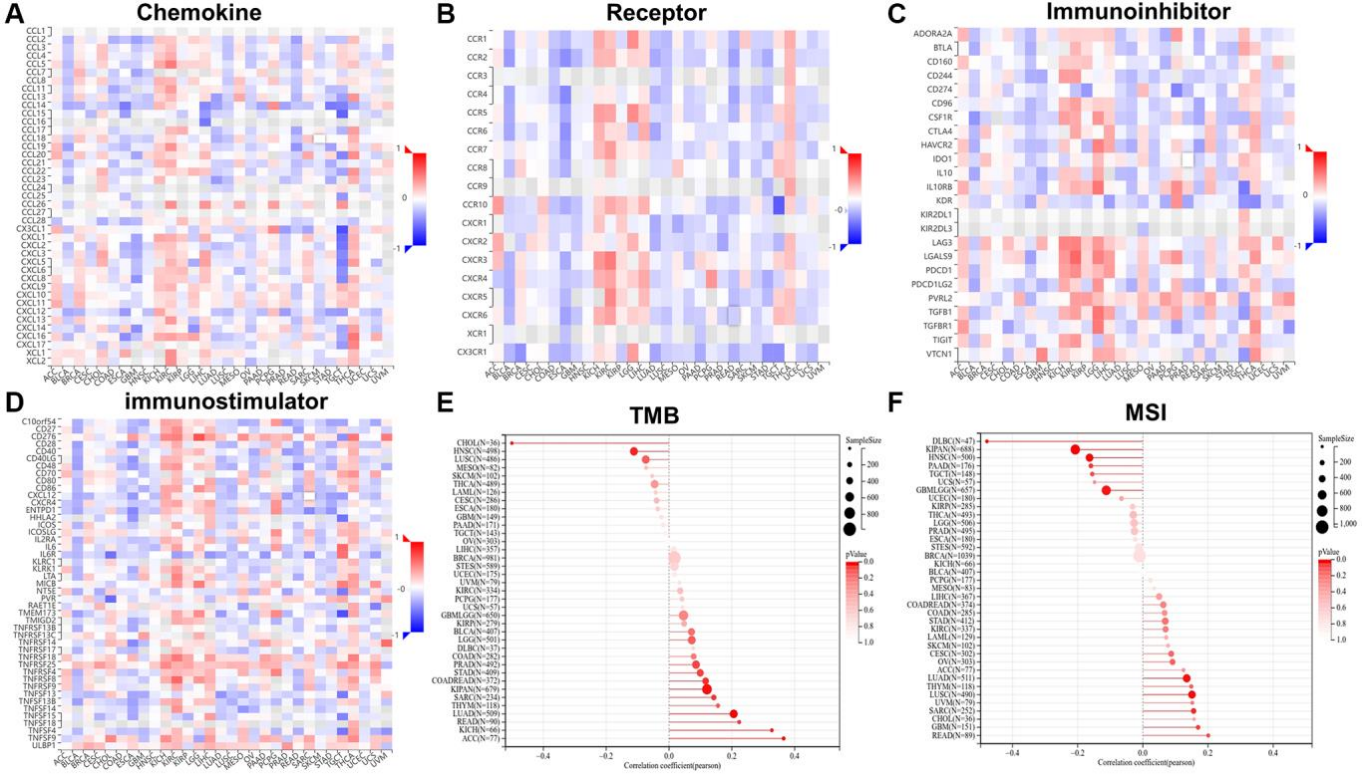
The relationship between *RCC2* expression and chemokine (**A**), receptor (**B**), immunoinhibitors (**C**), immunostimulators (**D**), tumor mutation burden (TMB) (**E**) and microsatellite instability (MSI) (**F**).

Next, we conducted a correlation analysis of *RCC2* with chemokine and receptor using TISIDB database, and the results displayed that *RCC2* expression was significantly distinct from that of chemokine in different tumors. We further revealed that the expression of *RCC2* was negatively related to immunomodulators in most cancers. In BRCA, LUAD, READ, ESCA and STAD, *RCC2* expression was negatively associated with chemokine and receptor. However, we discovered a positive correlation between *RCC2* and chemokine in KIRP, KICH, KIRC, THCA, and LIHC (Figure 8A, 8B). We further explored the correlation of *RCC2* expression and immunomodulator using the TSIIDB database. We observed that increased expression of *RCC2* in ACC, KICH, KIRC, and THCA positively correlated with most immunosuppressive markers (Figure 8C). Among the immunostimulatory factors, *IL6R*, *TNFRSF18*, *TNFRSF25,* and *ULBP1* were significantly associated with *RCC2* expression in the majority of cancer types. However, in ESCA, LUSC, LUAD, BLCA, and SKCM, *RCC2* expression level was negatively correlated with immunostimulatory marker genes (Figure 8D).

### Correlations between *RCC2* expression and MSI and TMB

TMB and MSI are genome-wide biological markers in predicting the treatment efficacy of immune checkpoint inhibitors (ICIs). We then investigated the connection between *RCC2* expression and MSI and TMB. The *RCC2* expression level was positively associated with TMB in LUAD, COAD, SARC, KIPAN, STAD, READ, ACC, and KICH and negatively related to TMB in HNSC and CHOL (Figure 8E). In GBM, SARC, LUAD, and LUSC, *RCC2* expression was positively related to MSI, while in LGG, KIPAN, HNSC, PAAD, and diffuse large B-cell lymphoma (DLBC), it was negatively related to MSI (Figure 8F).

### Protein-protein interactions network and enrichment analysis of *RCC2*

**Figure 9:**
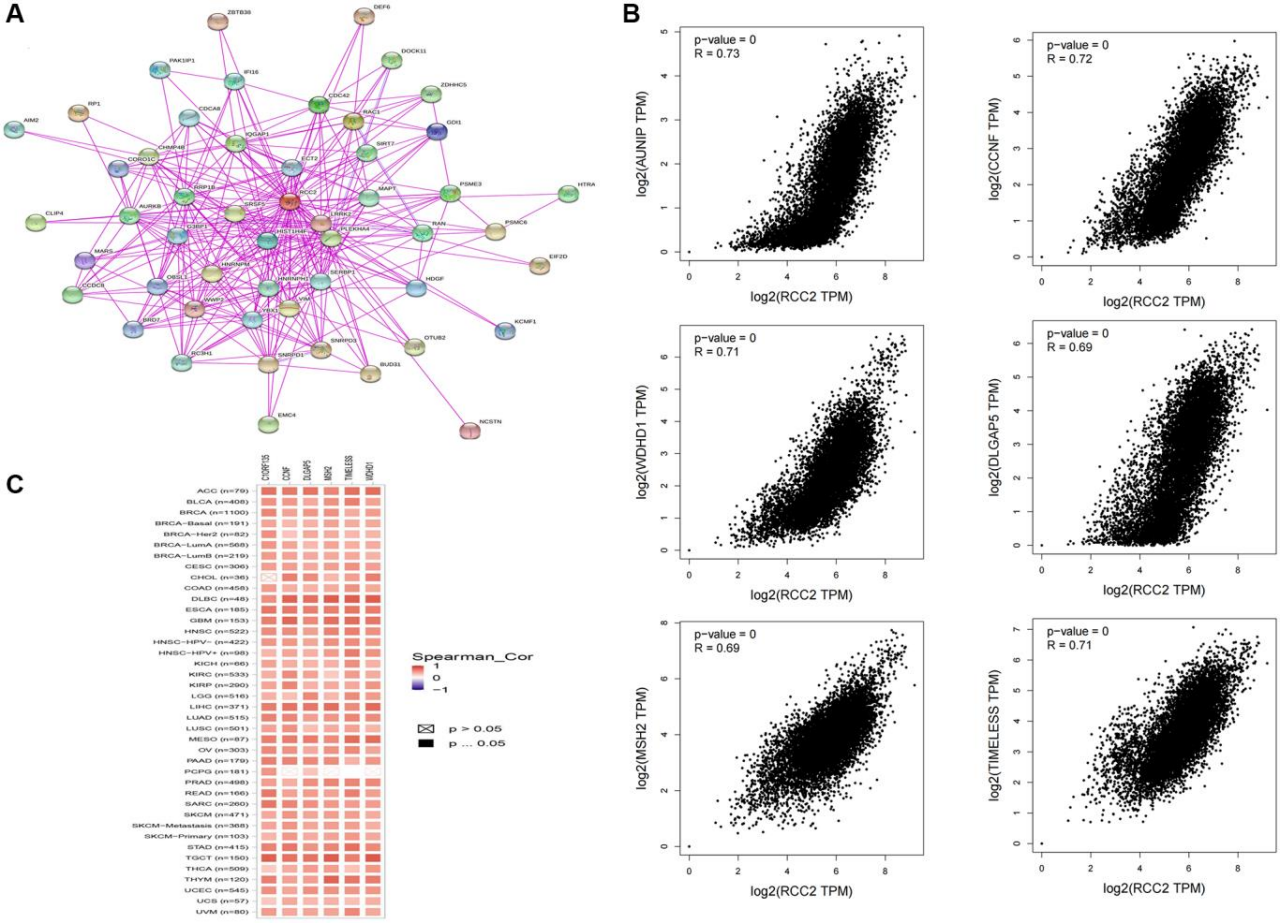
**Co-expression network of *RCC2* gene.** (**A**) *RCC2* binding proteins from the STRING website. (**B**) The “Expression analysis” module of GEPIA2 was used to obtain the 100 most closely related genes to *RCC2* expression, and the 6 most significantly related genes were AUNIP, CCNF, TIMELESS, WDHD1, MSH2, and DLGAP5. (**C**) Heat map of the correlation between *RCC2* and *AUNIP*, *CCNF*, *TIMELESS*, *WDHD1*, *MSH2,* and *DLGAP5* in most cancer types.

We used STRING and GEPIA2 to create a protein interaction network for *RCC2* and obtain *RCC2*-associated genes and performed enrichment analysis to investigate the potential molecular mechanisms of *RCC2* in cancer occurrence and development. At STRING, we built 50 *RCC2*-binding proteins based on the results of experiments, and we utilized GEPIA2 to identify the top 100 genes related to *RCC2* expression (Figure 9A). We analyzed the correlation of the top six-correlated genes and the results demonstrated that *RCC2* was highly related to *AUNIP*, *CCNF*, *TIMELESS*, *WDHD1*, *MSH2*, and *DLGAP5* (Figure 9B). Furthermore, in various cancer types, all six of these genes showed strong correlations (Figure 9C).

**Figure 10:**
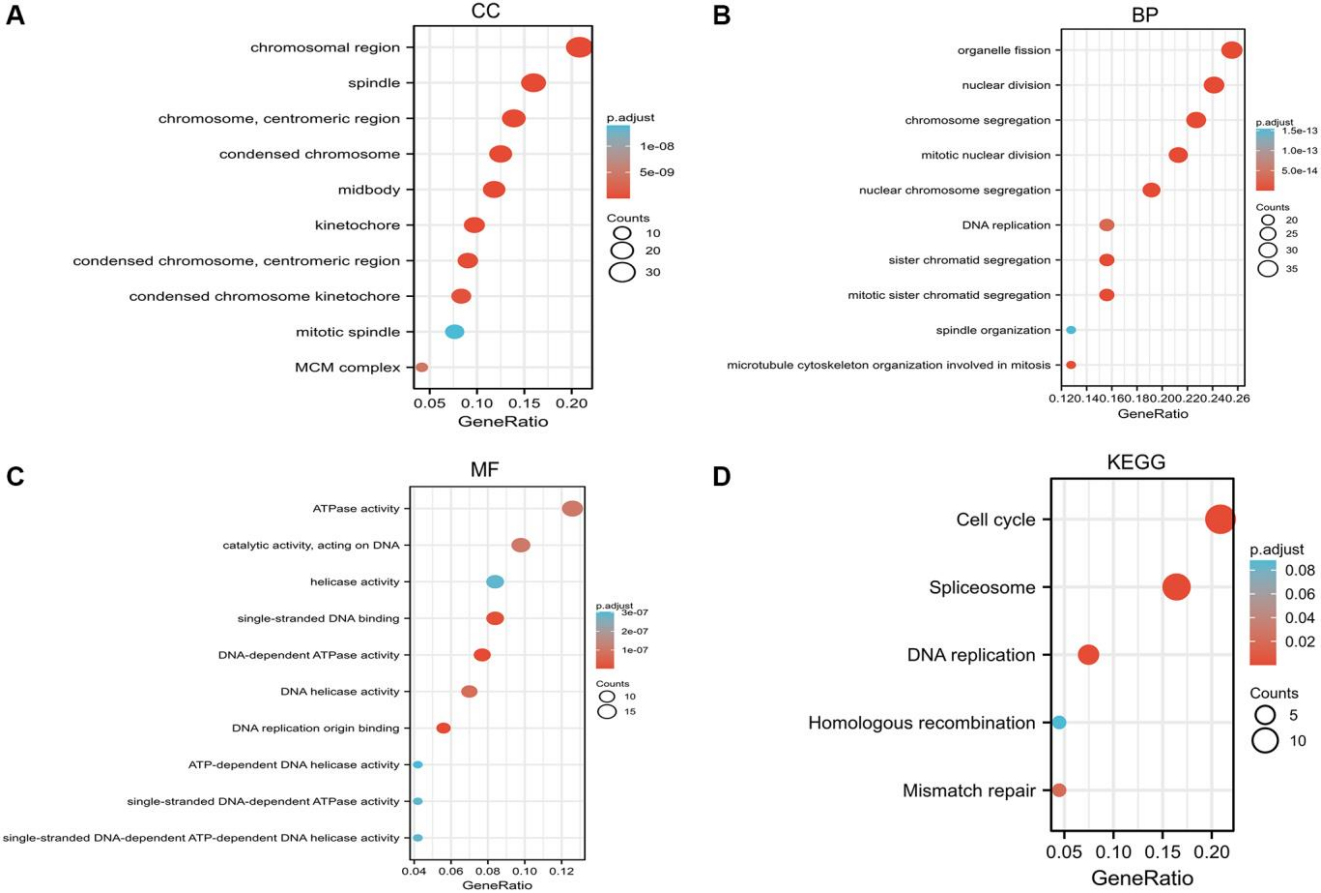
**Gene Ontology (GO) enrichment and Kyoto Encyclopedia of Genes and Genomes (KEGG) pathway analysis based on *RCC2* binding proteins and candidate genes.** (**A**) Cellular component (CC). (**B**) Biological process (BP). (**C**) Molecular function (MF). (**D**) KEGG pathway.

Next, we performed GO and KEGG functional enrichment analysis on *RCC2*. The cellular components (CC) are mainly in the chromosome, spindle, chromosomal region, and centromeric region (Figure 10A). The biological processes (BP) are mainly in chromosome segregation, organelle fission, and mitotic nuclear division (Figure 10B). In addition, the enriched molecular functions (MF) are related to DNA-dependent ATPase activity, single-stranded DNA binding, and DNA replication origin binding (Figure 10C). According to KEGG analysis, the main enrichment pathways are cell cycle, spliceosome, and DNA replication-related pathways (Figure 10D).

## DISCUSSION

*RCC2* is a cell cycle gene regulator associated with mitotic spindle formation, membrane dynamics, and cell cycle progression. It has a similarity to Ran’s known guanine nucleotide exchange factor. In addition, previous studies have shown that inhibition of *RCC2* blocks prophase cell activity, alters cell morphology, and increases apoptosis. *RCC2* is thought to be a regulator of cell migration and tumor metastasis. It was reported that *RCC2* overexpression promoted Wnt signaling pathway activation and induced EMT promoting an aggressive phenotype in breast cancer [4]. Lin et al. also showed that the upregulation of *RCC2* was involved in cigarette smoke extract (CSE)-induced autophagy and EMT in lung cancer cells [10]. In recent years, it has been shown that *RCC2* is a potent biomarker for hepatocellular and ovarian cancers [6, 11]. However, its role in pan-cancer is less understood.

In the present study, we analyzed the results by using data from the TIMER2.0 database showing that *RCC2* was overexpressed in most types of cancer relative to normal tissue, while it was only found to be downregulated in KICH. Differences in mRNA expression levels of the *RCC2* in various cancer types indicated its diverse roles in cancer development. Based on the CPTAC database, we discovered that the RCC2 protein was overexpressed in PAAD, BRCA, COAD, GBM, and OV tumors. Previous research revealed that the *RCC2* was elevated in breast cancer, ovarian cancer, and lung cancer [4, 5, 11]. These studies are consistent with our findings. Moreover, we found differential *RCC2* expression between different pathological stages in several tumors. In KIRC and KIRP, *RCC2* expression increased with stage, and the elevated *RCC2* expression was correlated with inferior survival, which suggested that *RCC2* may play a critical role in the carcinogenesis of KIRC and KIRP.

Moreover, we analyzed the prognostic value of *RCC2* in different tumor types. Our study found that *RCC2* overexpression was associated with LIHC, KIRP, KIRC, PAAD, and SARC with an inferior prognosis. However, in BRCA, ESCA, CESC, THYM, and STAD tumors, elevated *RCC2* was related to a favorable prognosis. KM survival analysis demonstrated that *RCC2* expression was correlated with unfavorable prognosis in lung adenocarcinoma. Previous research revealed that *RCC2* overexpression regulates the epithelial-mesenchymal transition and extracellular matrix remodeling of LUAD through JNK activation, thereby promoting the proliferation and metastasis of LUAD *in vivo* and *in vitro* [5]. These findings implied that *RCC2* may play an oncogenic role in many cancer types, and high expression of *RCC2* was correlated with a poorer survival outcome in hepatocellular carcinoma, which was in line with previous studies [6, 12]. However, some studies also found that *RCC2* played a role in promoting tumor growth and invasion in breast and esophageal cancers [4, 8]. The results of our analysis were inconsistent with previous studies, possibly due to different sample sizes in different databases. These results may suggest that *RCC2* plays different functions in different tumor types and could be used as a novel biomarker. However, the mechanism of *RCC2* in different tumors needs to be further explored.

Gene mutations are involved in tumor development, and gene mutation detection also plays a vital role in the treatment of cancer patients. There are few studies on *RCC2* gene alterations in pan-cancers. In our study, we identified the mutations as the main type of genetic alterations in *RCC2* in pan-cancers. We also explored that *RCC2* mutations were most frequent in stomach adenocarcinoma. However, we did not find a significant correlation between *RCC2* mutations and immunotherapy response.

The previous study revealed that CAFs have a role in promoting tumor progression [13]. CAFs can secrete large amounts of growth factors and pro-inflammatory cytokines and chemokines to assist immune escape [14]. As tumor cells continue to evade immune surveillance, immunosuppressive factors, like regulatory T cells, are upregulated in the TME. Our results showed that the *RCC2* gene was positively related to CAFs in most tumors and negatively associated with CAFs in TGCT tumors. It is suggested that the *RCC2* gene has a critical role of *RCC2* gene in regulating tumor immune microenvironment and the formation of CAFs, and the potential mechanism of *RCC2* regulation of CAFs needs further study. Targeting *RCC2* may be a prospective therapeutic approach for cancer.

Interactions between immune cells and other cells in the TME largely determine the intensity of anti-tumor immune response. According to the ESTIMATE analysis, the correlation of *RCC2* expression and immune-associated cell infiltration also occurred in pan-cancer. We observed a negative correlation of the stromal component and TME in 14 tumors, including GBM, LUAD, COAD, READ, BRCA, ESCA, STES, STAD, UCEC, HNSC, LUSC, SKCM-M, SKCM, TGCT, and BLCA. Besides, *RCC2* expression was significantly negatively associated with the immune score in 13 tumors, including GBM, CESC, LUAD, ESCA, STES, STAD, UCEC, LUSC, SKCM-M, SKCM, OV, SKCM-P, and BLCA. To date, there is still a gap in understanding the roles of *RCC2* in the immune system. The roles of *RCC2* in tumor immune microenvironment remains a new area worthy of further investigation.

Our correlation analysis also unraveled a close relationship between the *RCC2* level and the biological progression of various immune-related genes. In addition, we found that *RCC2* was co-expressed with MHC, immunosuppressive factors, immunostimulatory factors, receptors, and chemokines in most cancer types. *RCC2* showed a significant negative expression relationship with kinase insert domain receptor (KDR) in 20 of the 30 tumors. KDR regulates tumor angiogenesis and migration, previous work has demonstrated that KDR expression is strongly related to clinical outcomes and drug sensitivity in glioma and thyroid cancer [15, 16]. Meanwhile, IPS scores also play a critical role in predicting the response of cancer patients to ICI, and we can observe that high expression of *RCC2* in some cancers leads to a decrease in immune scores, which may lead to rapid tumor progression. In uroepithelial carcinoma, low expression of *RCC2* is more likely to produce an immune response to atezolizumab, which facilitates the clinical survival of patients. These findings implied that *RCC2* expression was closely related to immune infiltration of tumor cells and affected the immunotherapy respondence, providing a new target for improving immunotherapy in patients with various types of cancer. Meanwhile, TISIDB data showed considerable differences in *RCC2* expression in distinct cancer immune subtypes, implying that *RCC2* may serve as a valid biomarker for differentiating immune subtypes. In summary, *RCC2* was significantly expressed in many immune subtypes of tumors and associated with immune checkpoint genes and immune cell infiltration, suggesting *RCC2* may be involved in TME remodeling and affected the immunotherapeutic response of tumors.

TMB, which is a more tolerable predictor of the efficacy of ICIs, correlates with the efficacy of multiple tumor types of ICIs alone or in combination with two ICIs, and can be applied as a predictive marker of the immunotherapy efficacy [17, 18]. The increasing level of TMB represented higher possibility of neoantigens generation and the immune response rate [19]. Microsatellite instability (MSI) refers to variations in microsatellite length brought on by the insertion or deletion of microsatellite repeats in tumor tissues (MSI). MSI plays an essential role in the screening, treatment, and prognosis of cancers. MSI is a common phenomenon in tumors, which can predict the occurrence and progression of cancers and can also play an indispensable role in supporting diagnosis and drug guidance in different cancer types [20]. It has been shown that *RCC2* can be used as a prognostic marker in the MSI subgroup in colon cancer [21]. At present, in our results, we observed that *RCC2* expression was significantly related to TMB in COAD, READ, SARC, KIPAN, LUAD, STAD, READ, KICH, HNSC, and CHOL and was with MSI in GBM, LUAD, SARC, LUSC, GBMLGG, KIPAN, HNSC, PAAD, and DLBC.

There are still some limitations to the study. Firstly, most of the data were obtained from public databases, and different algorithms among different databases may result in bias in some findings, and further explorations are needed to validate the molecular mechanisms of *RCC2* in various tumors in the future. Secondly, the small sample size in several cancer types could also bring some bias to the analysis results.

## CONCLUSIONS

The present study revealed that *RCC2* was overexpressed in the majority of cancer tissues and affects clinical prognosis. The different outcomes of *RCC2* expression in diverse cancers prompted further exploration of its specific role in individual cancer. In addition, the expression of *RCC2* was related to immune cell infiltration, TMB, and MSI, and may influence the immunotherapeutic efficacy. Our comprehensive analysis highlighted the possibility of *RCC2* as a promising biomarker for diagnosis and therapy and may contribute to the precision and individualized cancer therapeutic strategies.

## MATERIALS AND METHODS

### Gene expression analysis

The Tumor Immune Estimation Resource (TIMER2) database is used to systematically analyze the immune infiltration of various tumors and to generate visual charts [22]. It has six advanced algorithms: TIMER, XCELL, CIBERSORT, CIBERSORT-ABS, TIDE, QUANTISEQ, EPIC and MCPCOUNTER. We download *RCC2* expression data in various malignancies and corresponding adjacent tissues through the “Gene_DE” module of TIMER2.0. *RCC2* expression level between adjacent normal and tumor tissues was assessed by Student’s *t*-test. The *p* < 0.05 was regarded as a statistically significant difference [23, 24].

UALCAN is an interactive web resource to perform analysis of cancer transcriptomic data from 31 cancer types in TCGA [25]. We used UALCAN to obtain the *RCC2* methylation data in different cancers. In addition, we also downloaded the mutation data of *RCC2* in pan-cancer from cBioPortal. Mutation data, including mutation type, alteration frequency, and mutated site information.

Gene Set Cancer Analysis (GSCALite) is a web server that integrates genomics and immunomics based on the TCGA database [26]. By downloading the *RCC2* expression and different tumor clinicopathological stages using the GSCALite website, we further used the Tumor-Immune System Interactions and Drug Bank (TISIDB) to explore the *RCC2* expression in various molecular subtypes of cancers [27].

### Gene alteration analysis

We used cBioPortal for Cancer Genomics tool to download the data on mutation type, alteration frequency, and copy number alteration (CNA) in pan-cancer patients [28, 29].

### Validation of protein expression in pancancer

Clinical Proteomic Tumor Analysis Consortium (CPTAC) integrates proteomic and genomic data and aims to identify and characterize the full range of proteins in tumor and normal tissues [30, 31]. The Human Protein Atlas project (HPA) has systematically generated antibodies against human proteins for profiling cancer-specific protein expression in tissues and cells [32]. We obtained differential RCC2 proteome expression and immunohistochemical (IHC) images in pan-cancer by integrating two databases.

### Survival prognosis analysis

Kaplan-Meier (KM) Plotter is a public platform that can quickly confirm the prognosis of disease [33]. We applied KM Plotter to explore the correlation between *RCC2* gene expression of relapse-free survival (RFS) and overall survival (OS) across all TCGA tumors. The hazard ratios (HRs) and 95% confidence intervals (CI) were calculated [34].

### Immune feature analysis

We investigated the association between *RCC2* expression and immune cell infiltration in all cancer types using the “immune correlation” module of the TIMER2 and the TISIDB database. Then, we further explored the relationship of *RCC2* expression with TMB and MSI using the Sangerbox 3.0 online website [35]. ESTIMATE scores presented an abundance of stromal and immune components. The correlation between *RCC2* expression and immunological and stromal scores was determined using the Spearman correlation analysis.

### Protein-protein interaction network and gene enrichment analysis

We then utilized the Search Tool for the Retrieval of Interacting Genes/Proteins (STRING) website for analysis of the protein interactions network [36]. Enter the *RCC2* gene and choose homo sapiens for the Organism. The basic settings are adjusted according to the following parameter information: The first shell’s settings for the network included “full STRING network,” “experiments only,” “meaning of network edges as evidence,” “Low confidence (0.150)”, and “no more than 50 interactors”.

Gene Expression Profiling Interactive Analysis (GEPIA2) is an online database, which was able to analyze RNA-seq expression data from a total of 8587 normal samples and 9736 tumor samples from TCGA and GTEx projects [37]. The top 100 *RCC2*-associated genes were obtained by GEPIA2. Pearson correlation analysis was then applied for *RCC2* and the top 6 selected genes, and correlation coefficients (R) and *p* values were calculated. A heat map of the correlation of *RCC2*-associated gene expression profiles in pan-cancer was downloaded using the TIMER2 database, containing *p*-values and partial correlations (cor) from the purity-adjusted Spearman rank correlation test.

We integrated 100 candidate genes and 50 binding proteins acquired from the two databases for the Kyoto Encyclopedia of Genes and Genomes (KEGG) pathway analysis and Gene Ontology (GO) functional enrichment. Enrichment analysis was performed using the “clusterProfiler” R package to obtain the results of gene set enrichment and to determine the potential functions and pathways of genes [38]. The “ggplot2” visualizes the top ten enrichment entries as bubble plots.

### Data availability statement

The datasets presented in this study can be found in online repositories. The names of the repository/repositories and accession number(s) can be found in the article/Supplementary Material.

## Supplementary Materials

Supplementary Figures

Supplementary Table 1
