# Supplementary material for: Comprehensive analysis of the expression profile and clinical implications of regulator of chromosome condensation 2 in pan-cancers
Source: Aging (Albany NY). 2022 Nov 27;14(22):9221–42. doi: 10.18632/aging.204403 (PMC9740375; doi:10.18632/aging.204403)
Supplement: Supplementary Figures [file aging-14-204403-s001.pdf]

SUPPLEMENTARY FIGURES

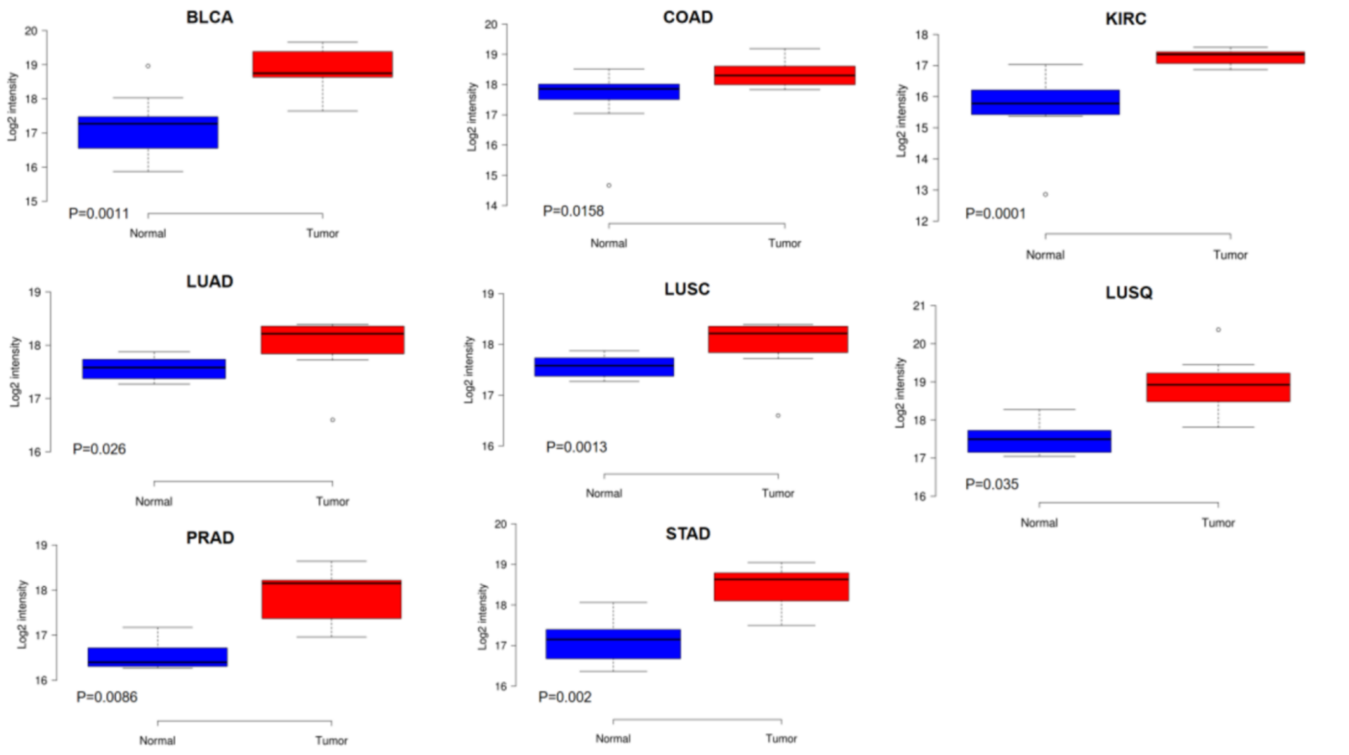

Supplementary Figure 1. RCC2 expression in proteomics data of 16 different cancer types. Only cancer types with $p < 0.05$ were illustrated.

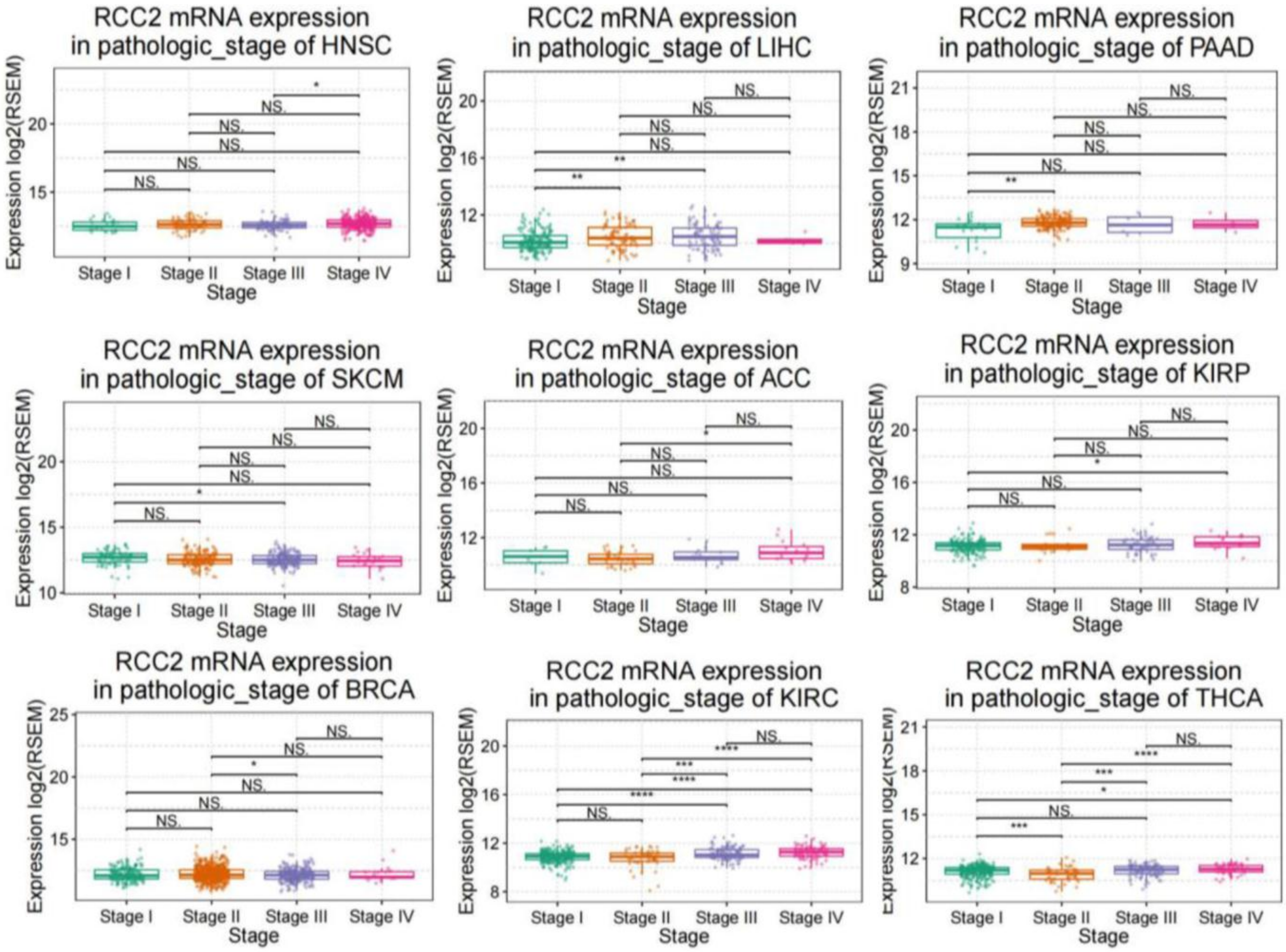

Supplementary Figure 2. ***RCC2*** **mRNA expression in pathologic stages of HNSC, LIHC, PAAD, SKCM, ACC, KIRP, BRCA, KIRC, and THCA.**

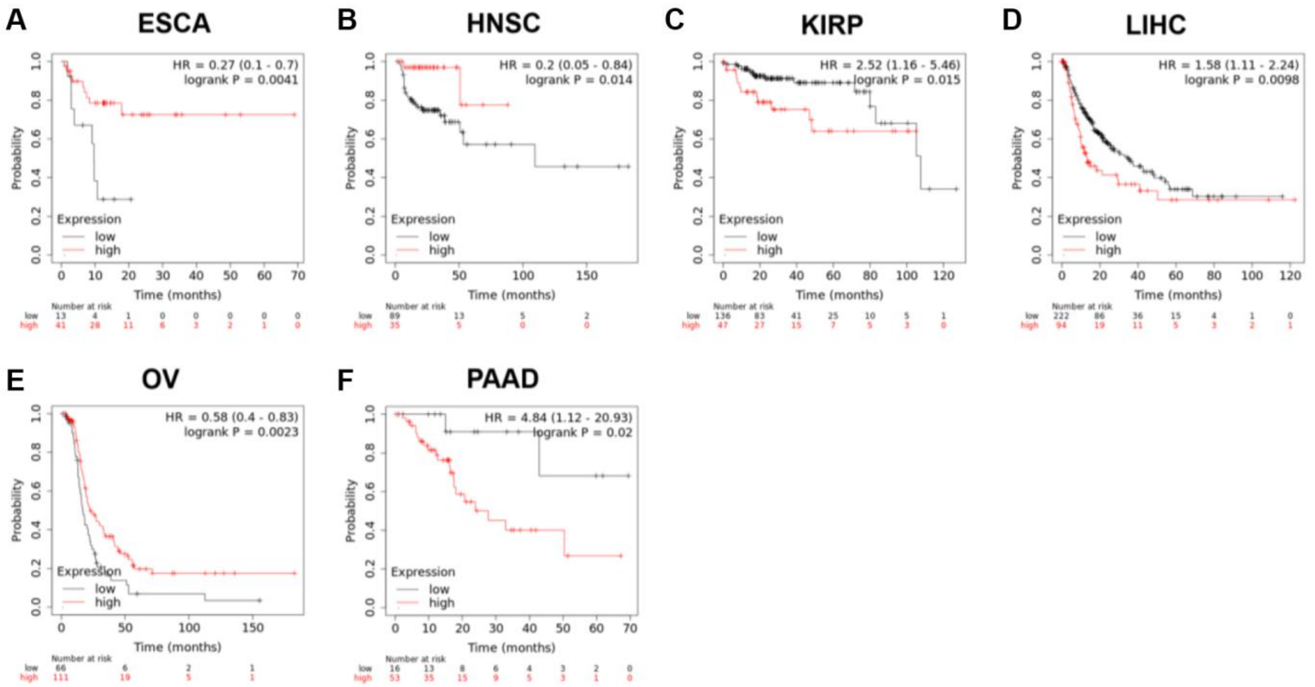

Supplementary Figure 3. (**A**–**F**) Relationship between *RCC2* expression and relapse-free survival (RFS) in TCGA pan-cancer. Only tumor types with log-rank $p < 0.05$ were displayed.

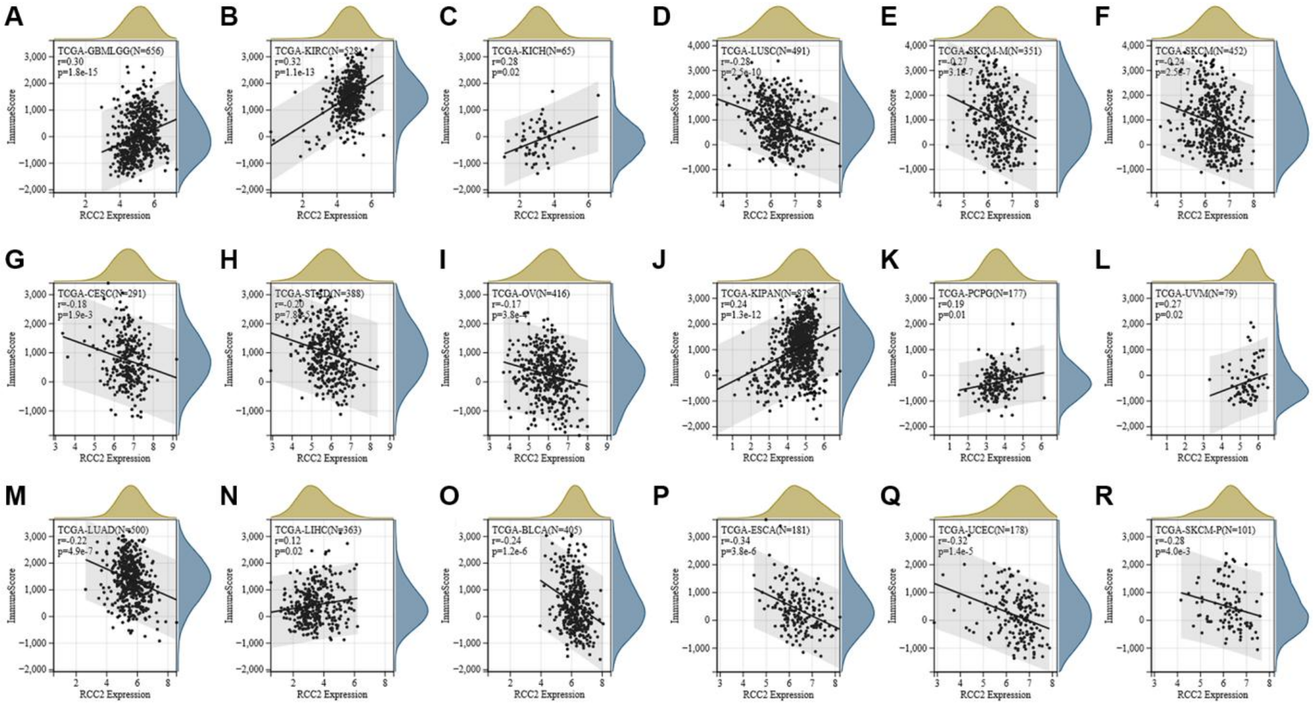

Supplementary Figure 4. (**A**–**R**) Correlation of *RCC2* expression with immune scores.

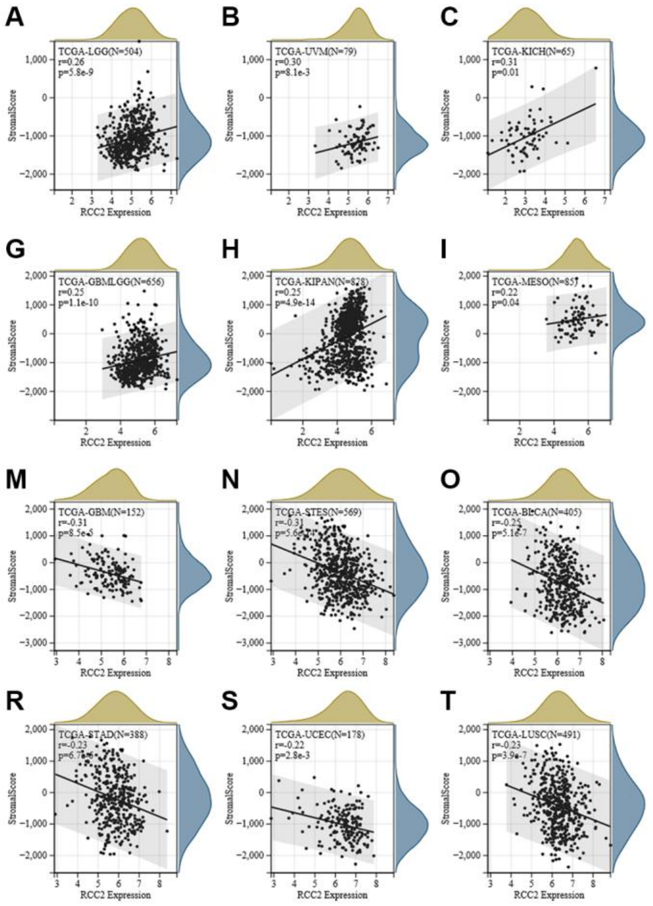

Supplementary Figure 5. (**A**–**T**) Correlation of *RCC2* expression with stromal scores.

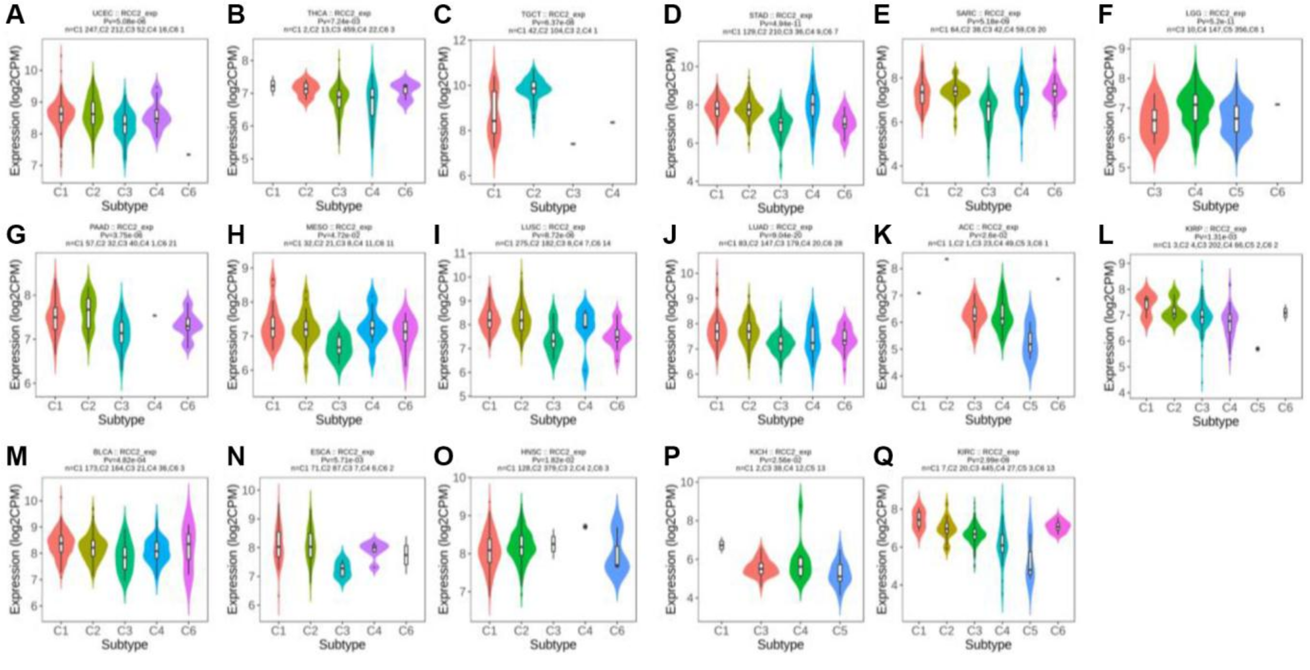

Supplementary Figure 6. Expression levels of *RCC2* in different types of tumor immune subtypes. (**A**) UCEC. (**B**) THCA. (**C**) TGCT. (**D**) STAD. (**E**) SARC. (**F**) LGG. (**G**) PAAD. (**H**) MESO. (**I**) LUSC. (**J**) LUAD. (**K**) ACC. (**L**) KIRP. (**M**) BLCA. (**N**) ESCA. (**O**) HNSC. (**P**) KICH. (**Q**) KIRC.

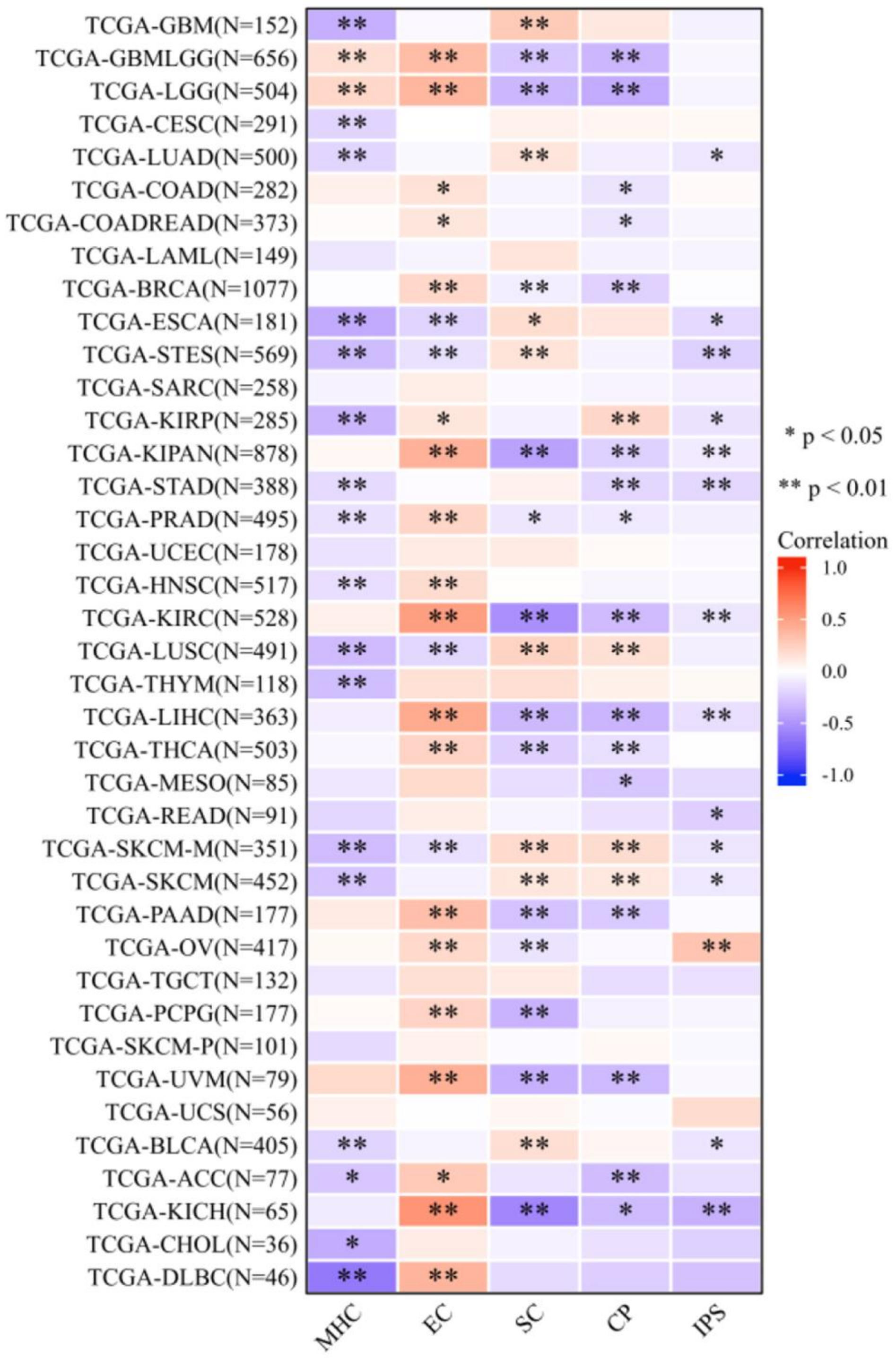

Supplementary Figure 7. Correlation of *RCC2* expression with MHC, EC, SC, CP, and IPS scores in pan-cancer. Abbreviations: MHC: major histocompatibility complex; EC: effector cells; SC: suppressor cells; CP: checkpoints; IPS: immunophenoscore.
