# Supplementary material for: Comprehensive analysis of the expression profile and clinical implications of regulator of chromosome condensation 2 in pan-cancers
Source: Aging (Albany NY). 2022 Nov 27;14(22):9221–42. doi: 10.18632/aging.204403 (PMC9740375; doi:10.18632/aging.204403)
Supplement: Supplementary Table 1 [file aging-14-204403-s002.pdf]

SUPPLEMENTARY TABLE

Supplementary Table 1. *RCC2* expression between responders and non-responders of immunotherapy among different cancer types.

Cancer type	Group	Drug	#Res	#NRes	Log2 (fold change)	*P* value
Melanoma	all	Anti-PD-1 (pembrolizumab and nivolumab)	14	12	−0.044	0.84
Melanoma	MAPKi	Anti-PD-1 (pembrolizumab and nivolumab)	6	5	0.065	0.981
Melanoma	non-MAPKi	Anti-PD-1 (pembrolizumab and nivolumab)	8	7	−0.122	0.952
Urothelial cancer	all	Anti-PD-L1 (atezolizumab)	9	16	0.409	0.347
Urothelial cancer	smoking	Anti-PD-L1 (atezolizumab)	5	9	0.481	0.823
Urothelial cancer	non-smoking	Anti-PD-L1 (atezolizumab)	4	7	0.323	0.911
Melanoma	all	Anti-PD-1 (nivolumab)	26	23	0.032	0.945
Melanoma	NIV3-PROG	Anti-PD-1 (nivolumab)	15	11	−0.247	0.907
Melanoma	NIV3-NAIVE	Anti-PD-1 (nivolumab)	11	12	0.355	0.88
Clear cell renal cell carcinoma (ccRCC)	all	Anti-PD-1 (nivolumab)	4	8	0.968	0.554
Clear cell renal cell carcinoma (ccRCC)	VEGFRi	Anti-PD-1 (nivolumab)	2	0	0	1
Clear cell renal cell carcinoma (ccRCC)	non-VEGFRi	Anti-PD-1 (nivolumab)	2	8	1.241	0.618
Urothelial cancer	all	Anti-PD-L1 (atezolizumab)	68	230	0.208	**0.00133**

#Res represents the number of responders; #NRes means the number of non-responders.
